# Are current practices of yak herdsmen adequate to combat *Coenurosis* in Laya Bhutan?

**DOI:** 10.1002/vms3.466

**Published:** 2021-03-23

**Authors:** Yeshi Wangdi, Kesang Wangchuk

**Affiliations:** ^1^ National Highland Research and Development Center Ministry of Agriculture and Forest Bumthang Bhutan; ^2^ International Center for Integrated Mountain Development Kathmandu Nepal

**Keywords:** coenurosis, gid, herdsmen, *Taenia multiceps*, watchdogs, yak

## Abstract

**Background:**

Coenurosis, known commonly as gid, is caused by a tapeworm *Taenia multiceps*. It is a disease of small ruminants globally but also occurs in large ruminants, especially in yak in the Himalaya. Gid is a pathological condition in young yaks, mostly below 3 years of age. The infected animal displays a circling movement with the head tilting towards the location of the cyst of a tapeworm on the cerebral surface of the brain.

**Objective:**

We conducted a study with the primary aim to gain an insight into yak herdsmen's practices to manage Coenurosis in the Laya administrative block of Bhutan.

**Methods:**

All seven villages of Laya were included for sampling. Seventy‐five out of 182 households owned yaks, and 54 yak‐owning households were selected randomly. The government livestock officials of nine yak‐rearing highland districts were also included in the study. A cross‐sectional study was conducted based on a questionnaire survey and focussed mainly on herdsmen's practices to manage gid. Two sets of questionnaires were used for yak herders and livestock officials. Each predesigned questionnaire was semi‐structured and consisted of both open– and closed‐ended questions.

**Results:**

The study revealed gid as a major cause of yak mortality. Gid occurred more in winter during migration and most herders lost one to three yaks annually. Herdsmen kept an average of two watchdogs and dewormed them once annually. Similarly, calves were also dewormed once annually. The carcasses of dead yaks were fed to dogs. Livestock officials were optimistic about controlling the disease in the future, despite the yak areas being difficult to access. Most herders had not attended the gid awareness programme. The animal health worker visited herds once annually. In absence of animal health workers, most herdsmen resorted to different practices to treat affected yaks –the most common practice being surgery. Gid was mentioned to harm herdsmen's economy.

**Conclusions:**

The study concluded that for effective management of gid in Laya, livestock agencies must create more awareness on gid, increase the frequency of visits by animal health workers to yak herds, and increase the frequency of deworming of watchdogs and calves.

## Graphical abstract

Gid is an economically important disease in yaks. It affects the livelihood of herders in the mountains.

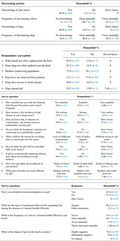

## 1 INTRODUCTION

Coenurosis, known commonly as gid, is a disease of the brain and spinal cord caused by the larval stage of the tapeworm *Taenia multiceps* (FAO, 2020; Garcia & Brutto, 2012). The disease occurs in small ruminants, mainly in the sheep–farming regions of Europe, the Americas, Africa and Asia (Lescano & Zunt, 2013). The larval stage of the tapeworm is found in the intermediate hosts. The clinical disease is rare in cattle (FAO, 2020), although it has been diagnosed in different countries (Varcasia et al., 2013). The syndrome of gid is produced by a lesion formed in the central nervous system of an infected animal (Constable et al., 2017). The risk of human health and economic losses of gid disease in small ruminants, caused by discarding of infected meat, have been reported (Shiferaw & Abdela, 2016; Shiferaw, 2018).

In the Himalayan country of Bhutan, gid occurs in large ruminants, mainly yak (*Bos grunniens*). Gid is reported as a pathological condition in young yaks, mostly below 3 years of age (Dorji et al., 2003; NCAH, 2015, 2016; Palden, 2016; Samdrup, 1992; Wangdi, 1996). *T. multiceps* is transmitted between dogs and domestic herbivores (Güçlü et al., 2006). The infected yak displays a circling movement with head tilting towards the location of the cyst of a tapeworm on the cerebral surface of the brain. Gid is a serious disease in yaks, reported since the 1950s and prevails across the yak‐rearing regions. It is also a zoonotic parasitic disease and shares the same environment along with another important parasitic zoonosis cystic echinococcosis. Watchdogs are the main definitive hosts, responsible for the transmission of gid to yaks (NCAH, 2016), although Varcasia et al. (2015) also discussed the role of red foxes in the epidemiology of *T. multiceps* (Varcasia et al. (2015). On the contrary, domestic cats and wild felids are not considered suitable definitive hosts. Yak herdsmen keep watchdogs to herd and guard yaks from wild predators. Coprological examination of watchdogs’ faeces confirmed the presence of eggs of *T. multiceps* (NCAH, 2015, 2016). Acharya et al. (2016) detected the eggs of *Taenia spp*. and speculate on the chance that the *Taenia spp*. might represent pass‐through (i.e. not parasitic) because of direct contact with herd dogs and contamination of grazing pastures.

In Bhutan, the Gid Prevention and Control Programs were initiated in major yak‐rearing areas in the 1950s. The programmes provide a more strategic and effective approach constituting social, cultural and management aspects of dealing with Coenurosis (DoL, 2016). Despite the concerted efforts, gid remains persistent and continues unabated, causing huge economic losses to yak herders in most yak‐rearing regions of northern Bhutan. The situation is aggravated by the lack of studies to evaluate the extent of adoption of practices recommended for gid control. The knowledge of the adoption of practices is important, as it forms a basis for planning future management interventions. Information on current practices helps understand the proportion of yaks in a given population that is infected by gid. Hence, it not only helps in estimating resources needed to treat infected yaks but is also useful to agencies responsible for planning and providing health services. Therefore, this study aims to gain an insight into practices adopted by yak herders to control gid in the Laya administrative block of Bhutan.

## 2 MATERIALS AND METHODS

### 2.1 Study area

**FIGURE 1:**
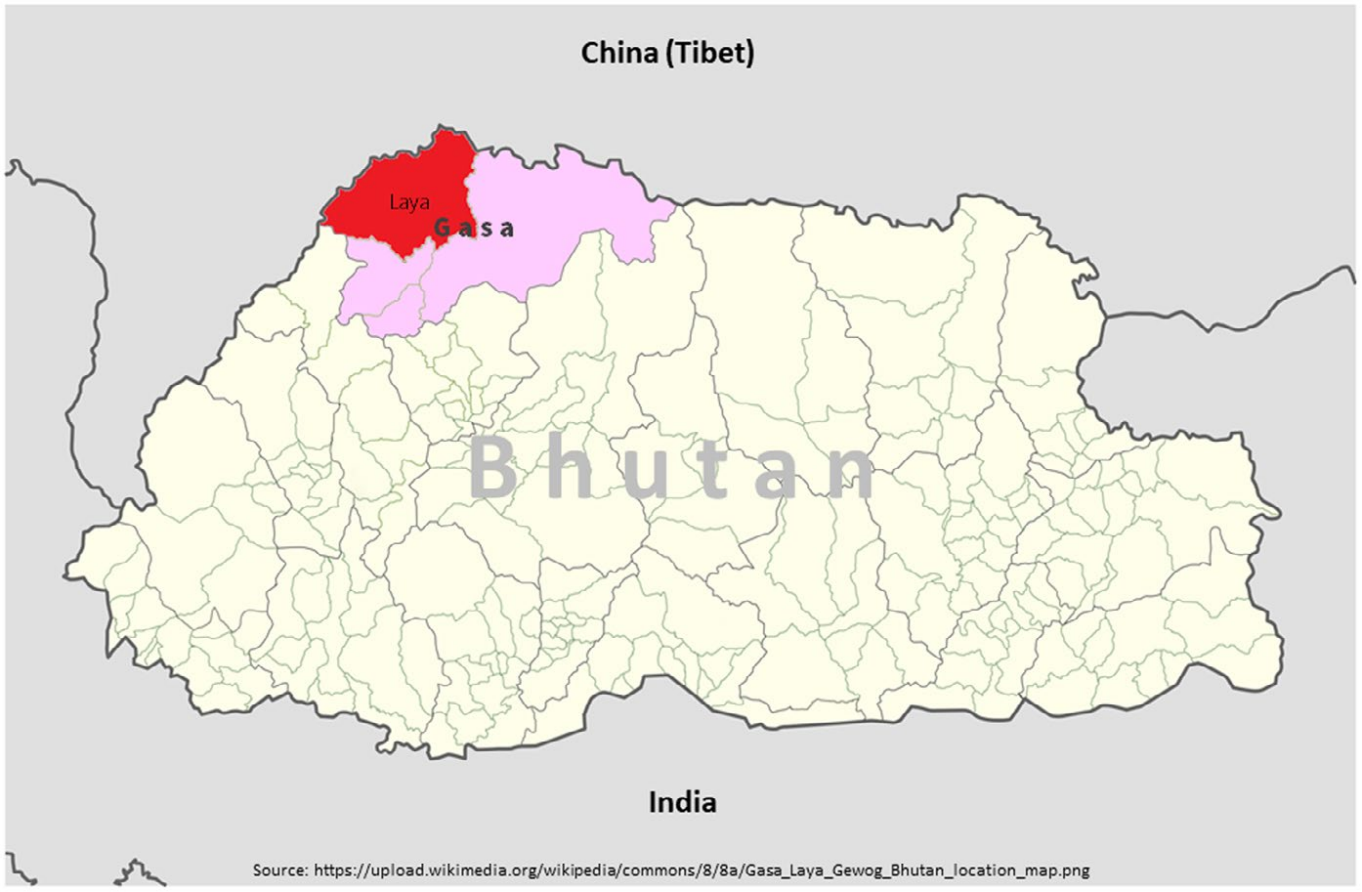
Location map of study site in Laya under Gasa district

The district of Gasa (27.8983°N, 89.7310°E) in Northern Bhutan was selected for the study (Figure 1). Gasa lies in the extreme northwest, bordered by Punakha district in the southeast, Thimphu district in the southwest, Wangdue district in the east, and Tibet (China) in the north. It has a total area of 3,117.74 sq. km, covering 11% of Bhutan's total area. The altitude ranges from 1,500 to 4,500 m above sea level (National Statistics Bureau, 2010). Among the yak‐rearing districts, Gasa was considered a reliable district for information on gid and was selected for the field survey, as it has a greater number of cases of yaks affected by gid. The district has two major yak‐rearing administrative blocks: Laya and Lunana. Laya (28.0636°N, 89.6828°E) was selected as the main study site, because of a relatively high population of 3,512 yaks (DoL, 2018) and easy access to villages. The area of Laya is approximately 981.5 sq. km (National Statistics Bureau, 2005). Laya experiences moderately cold and wet summer (June–August), followed by freezing winter (November–April). Herdsmen practice transhumant agro‐pastoralism and migrate to lower elevation in winter and vice versa in summer.

### 2.2 Sampling and study design

All seven villages of Laya were included for sampling. The total number of households was 182, but only 75 households owned yaks. Yak herds with each herd having more than 10 yaks were considered for the study. Out of 75 households, 54 had herd size above 10 yaks and were selected for the survey. The survey also included livestock officials of yak‐rearing districts. Altogether, a cross‐sectional study was conducted, using two sets of questionnaires for yak herders and livestock officials. The focus of the questionnaire‐based survey was on the prevalence of gid disease.

The predesigned questionnaires were semi‐structured and pre‐tested on three herders during the Third Royal Highland Festival in 2018. Where required, the questionnaires were amended and finalised. The enumerator conducted face–to–face interviews with the respondents engaged actively in managing yak herds. The questionnaires consisted of both open– and closed‐ended questions. The questions also probed to extract vital information and causes of gid prevalence, besides emphasising yak and dog management. Herdsmen were requested to express their expectations from the government to control the disease. For livestock officials, the questions were framed to seek their perceptions on gid prevalence, the effectiveness of the gid control programme, reasons for failure to control gid, gid as an economically important disease, and types of herders affected by gid.

### 2.3 Data analysis

Data were analysed by SPSS version 24 (IBM, 2004). Descriptive statistics were used to summarise data and generate estimates in percentages. Microsoft Excel 2019 was used to prepare graphs for presenting results.

## 3 RESULTS AND DISCUSSION

### 3.1 Size of yak herd and causes of yak mortality

**FIGURE 2:**
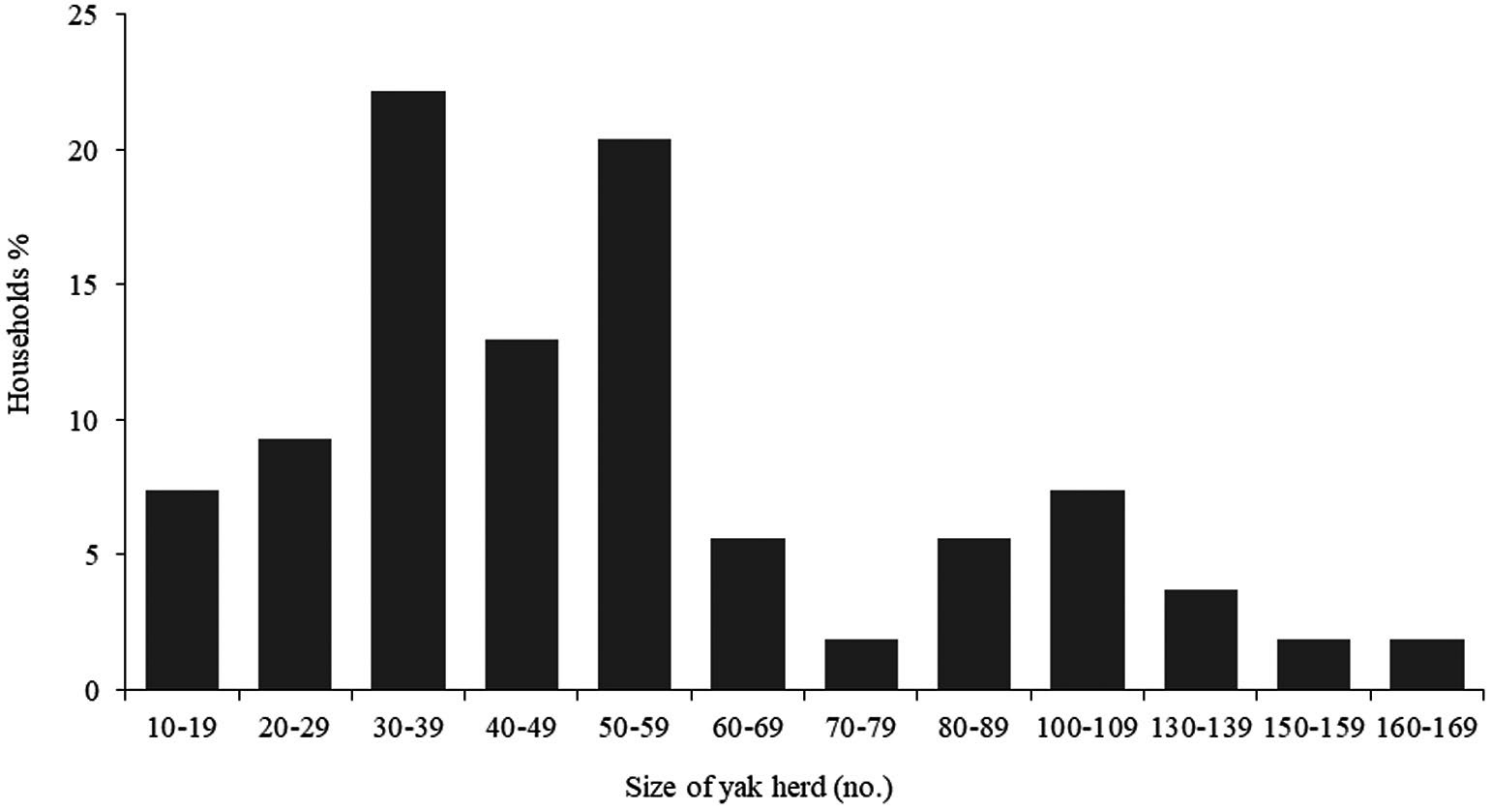
Categories of herd size in Laya subdistrict

Most households had an average herd size of 30–50 yaks per household (Figure 2). The herd size is far below the national average of 66 yaks per household (Wangdi, 2016), but corresponds to the average size of 42 animals in Central Bhutan (Dorji, 2000). Only a few households had bigger herds.

**FIGURE 3:**
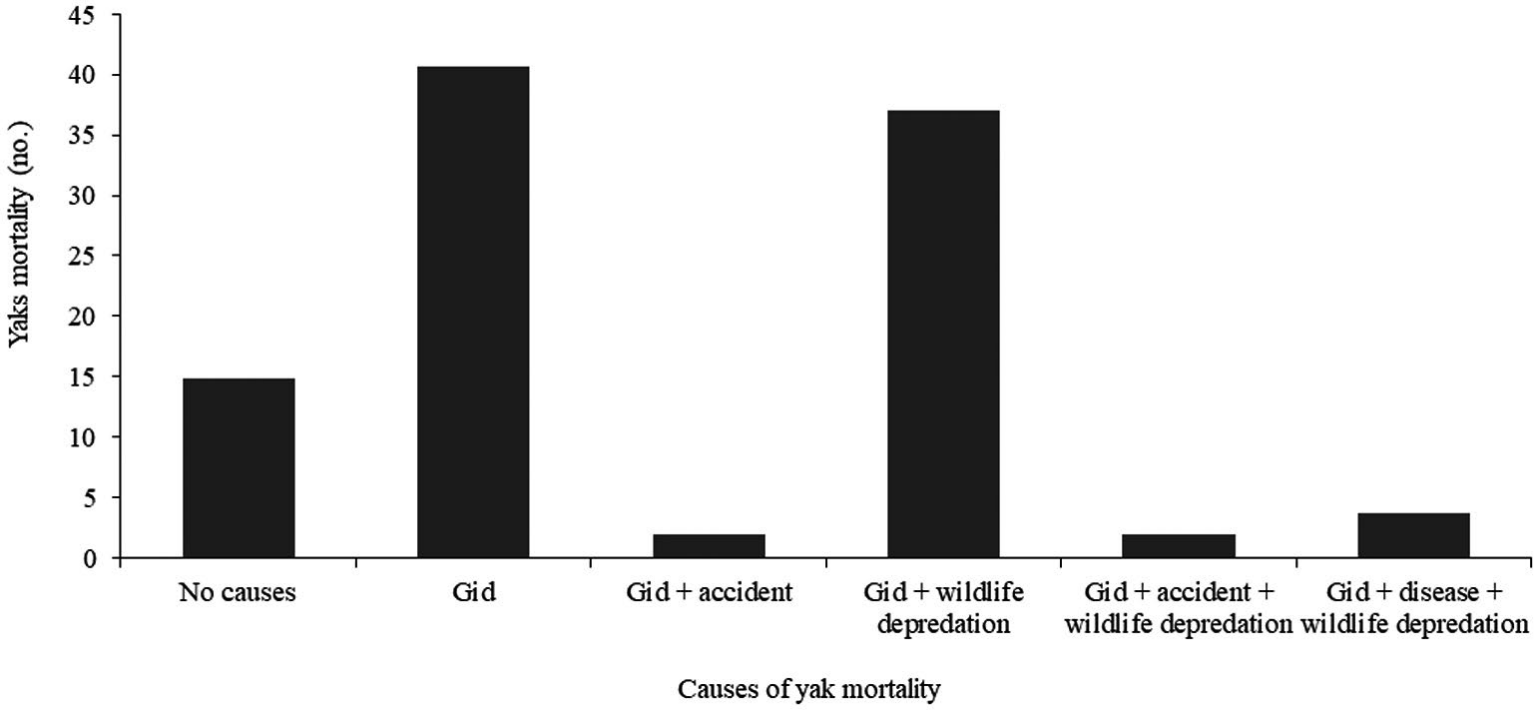
Common causes of yak mortality in Laya subdistrict

**FIGURE 4:**
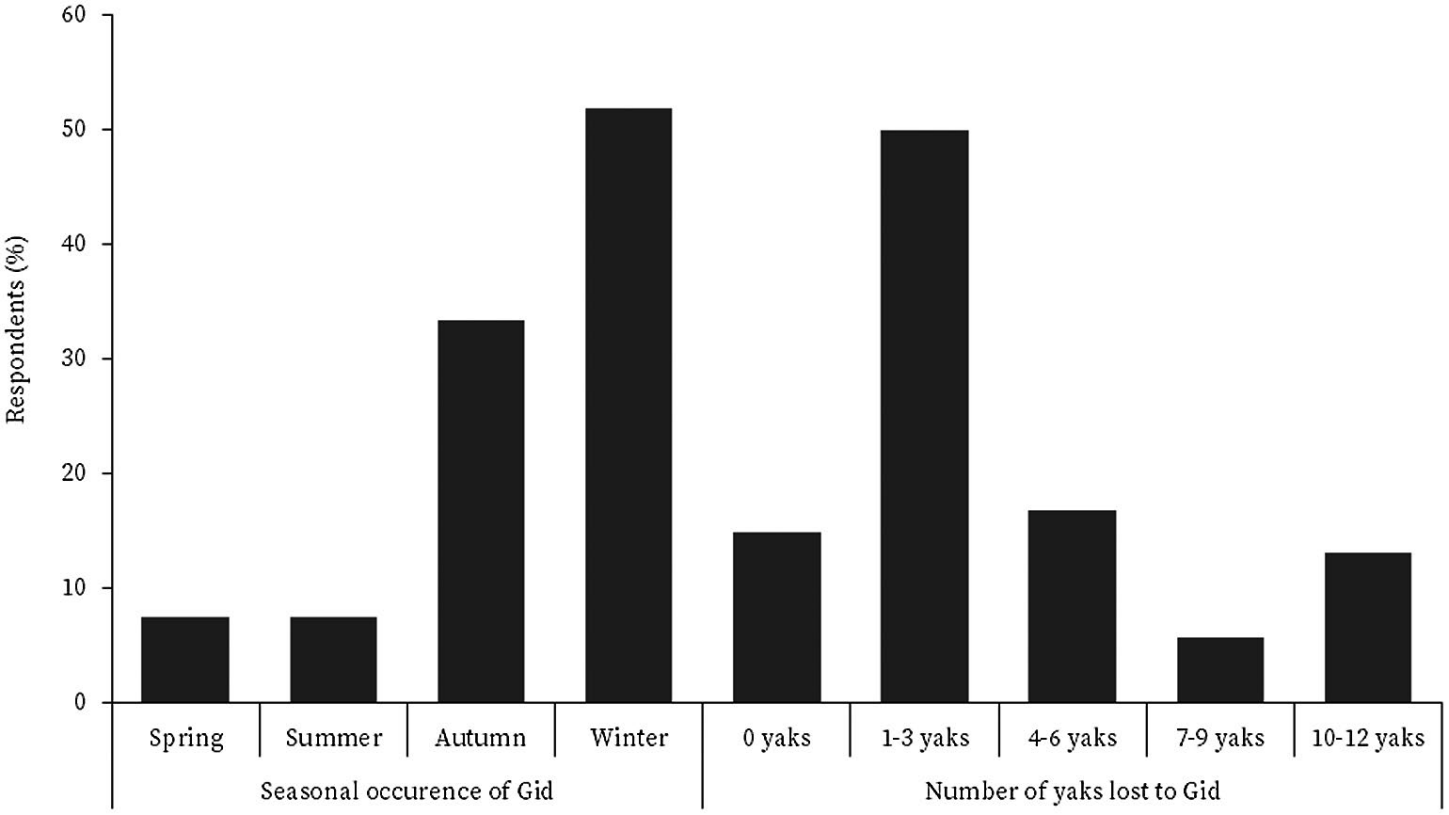
Seasonal occurrence of Gid and number of yaks lost to Gid

There were several causes of yak mortality in Laya; however, gid was a major cause, followed by a combination of gid and wildlife predation (Figure 3). Among wildlife, bears and snow leopards are the main predators of yaks (Sangay & Vernes, 2008). Gid was mentioned to occur more in winter during migration; this contradicts the report of Sharma et al. (1998) that gid has no significant seasonal variation. Affected yaks could likely have died in spring, as reported by Gyamtsho (2000) that gid leads to death in spring when yaks are very weak due to severe forage scarcity. The cyst of *T. multiceps* has been reported to mature in approximately 8 months when infected yak shows nervous symptoms (Abera et al., 2016). Therefore, the maximum occurrence of gid in winter (Figure 4) suggests that yaks are infected in summer while grazing on pastures contaminated with eggs of *T. multiceps*. Infection in summer could be attributed to the ecological variable (Shiferaw et al., 2016), mainly the rainfall that may have facilitated the spread of faeces of dogs and wild canids over the meadows, increasing the chances of infection by *T. multiceps*. Hashim et al. (2000) attribute increased occurrence of gid during the rainy season to the spread of contaminates of canids.

Over half of the total households had lost at least one to three yaks annually to gid (Figure 4), suggesting that highland communities continue to lose yaks to gid annually. Gid was a major cause of yak mortality in the late 1990s (Gyamtsho, 1996), and it continues unabated in Bhutan even today. In this study, the gid frequency of over 32% (Figure 3b) in calves below 3 years in Laya is almost two times greater than the frequency of over 17% in 2015 (NCAH, 2016). Although the Ministry of Agriculture and Forests of Bhutan made a renewed approach to combat gid by launching a National Gid Disease Prevention and Control Plan during the First Royal Highland Festival in 2016 in Laya, it is yet to be effective. Efforts were made to carry out awareness campaigns for yak herders but have been futile, which could be attributed to the remoteness of yak herders and poor coordination amongst development agencies.

### 3.2 Prophylactic measures of herders to combat gid

The majority of households kept an average of two dogs per household. It is customary for yak herders to keep dogs for herding and guarding yaks. Dogs are also kept near the transit camps to guard properties when herders are away. The tradition of dog keeping may have been useful but it has contributed to spreading gid, which threatens the livelihoods of yak herders. Similar incidence has been reported in the sheep‐rearing areas, where the presence of shepherd dogs on grazing land and paddocks, greatly contributed to the existence of Coenurosis (Abera et al., 2016).

A large majority of households dewormed their dogs (Table 1), indicating herders’ understanding of dogs as a definitive host of gid. However, a vast majority of households dewormed dogs once annually, against the recommended annual deworming frequency of six times at 2‐monthly intervals from February (NCAH, 2016). Deworming is done by administering anthelmintic drug Praziquantel (5 mg per kg body weight at 2 months interval) in dogs to rid them of internal parasites, mainly the tapeworm (NCAH, 2016). The continued prevalence of gid suggests that a single deworming in a year is ineffective, and also due to the use of Praziquantel alone. Further, the efficacy of quarterly deworming of dogs to control gid (Alemu et al., 2016) may be debatable, as the prepatent period of *T. multiceps* is of around 5–8 weeks (Del Brutto, 2014). The lack of good hygiene may also have facilitated the spread of gid, as poor hygienic handling of contaminated meat and infected animals is common among yak herders. Herders also dewormed yak calves, but the deworming frequency was once annually, against the recommended annual deworming frequency of two times with the first deworming in March–April and second in October–November (NCAH, 2016). A strategy to deworm yak calves is to improve growth and herd productivity. Deworming is found to improve milk quality in dairy cattle (Thapa Shrestha, et al., 2020). Calves are dewormed with Albendazole (7.50 mg per kg body weight), a medicine effective in controlling immature or larval stages of the tapeworms (NCAH, 2016). The rationale behind deworming calves is because calves are infected easily by the larva of tapeworm, which caused huge calf mortality in the 1960s and 2003–2013 (NCAH, 2016). Similar to dog deworming, a single annual deworming of calves appeared ineffective. The ineffectiveness of deworming could also be attributed to the use of albendazole alone.

**TABLE 1:** Deworming and frequency of deworming of calves and dogs in Laya sub‐district

Deworming activity	Household %		
Deworming of yak calves	Yes	No	Don't know
	88.9 (*n* = 48)	11.1 (*n* = 6)	0
Frequency of deworming calves	No deworming	Once annually	Twice annually
	11.1 (*n* = 6)	66.7 (*n* = 36)	22.2 (*n* = 12)
Deworming of dogs	Yes	No	Don't know
	86.0 (*n* = 44)	14.0 (*n* = 10)	0 (*n* = 0)
Frequency of deworming dogs	No deworming	Once annually	Twice annually
	14.0 (*n* = 10)	48.0 (*n* = 26)	38.0 (*n* = 18)

Table 2 presents respondents’ perception of dogs and wild canids as transmitters of gid. A vast majority of households agreed that dogs spread gid, which explains why most households dewormed dogs. However, most households also agreed to dogs having easy access to carcasses. Probably, out of ignorance, it is common among herders to feed the infected yak skull to dogs or leave the skull to be eaten by stray dogs and wild canids. Thus, the faeces of dogs and wild canids contaminate the environment. Abera et al. (2016) found dogs to maintain *C. cerebralis* –*T. multiceps* life cycle when they are frequently fed with heads of butchered animals not treated for parasitic diseases. A similar practice has also been reported in sheep farming areas (Scala & Varcasia, 2006).

**TABLE 2:** Respondents’ perception of dogs and wild canids as transmitters of Gid

Respondents’ perception	Household %		
	Yes	No	Do not know
1. Wild canids are often sighted near the herd	87.0 (*n* = 47)	13.0 (*n* = 7)	0
2. Stray dogs are often sighted near the herd	85.2 (*n* = 46)	14.8 (*n* = 8)	0
3. Herders control dog population	75.9 (*n* = 41)	24.1 (*n* = 13)	0
4. Dog faeces are removed from pasture	22.2 (*n* = 12)	77.8 (*n* = 42)	0
5. Dogs have access to dead carcass	84.0 (*n* = 45)	16.0 (*n* = 9)	0
6. Dogs spread gid	92.5 (*n* = 50)	1.90 (*n* = 1)	5.60 (*n* = 3)

Although most households felt the need to control the dog population, a large majority of households never cleaned and removed dog faeces from pastures. Despite the repeated reminders on the importance of removing faeces, herders never heed the advice of livestock development workers. It is understandable because meadows are vast and spread across rugged terrain and it is difficult for herders to go out looking for faeces that are scattered all over the vast meadows. Also, due to a busy herding schedule, herders do not find time for cleaning vast meadows. Meadows are also frequented by stray dogs and wild canids, suggesting that they also contaminate pastures with their faeces. Varcasia et al. (2004) reported small predators like foxes to feed on dead animals in pastures with a likelihood of contaminating pastures with their faeces.

### 3.3 Perceptions of livestock officials on Coenurosis

The perceptions of livestock officials of yak‐rearing areas of Bhutan are presented in Table 3. The livestock officials, mainly the District Livestock Development Officials, have a vital role to enhance livestock production in the districts through the dissemination of improved practices of livestock husbandry and control of livestock diseases. The survey revealed over half of the livestock officials (55%) being satisfied with the National Gid Prevention and Control Program. Although the programme may be perceived as effective, the ground reality seems to contradict, as indicated by gid being serious in the yak‐rearing districts according to half of the officials (50%). It reflects a challenge that the government is facing to contain the disease effectively. As an ultimate measure to combat the disease in absence of veterinarians, the majority of livestock experts (39%) are aware of herders treating affected yaks in traditional ways. Such traditional treatment practices were perceived by a large majority of officials (67%) as scientifically incorrect. Difficult access to yak areas was the main reason for not being able to control the disease effectively, according to 67% of officials. Such a challenge is also found in the Asian highlands where yak is reared (Kreutzmann, 2002; Long et al., 2008). However, most officials (56%) were optimistic about controlling Coenurosis in the future, as 94% of officials view it as an economically important disease, affecting the herders’ livelihood (72%) through the death of calves. A large majority of officials (72%) mentioned gid to affect large herds.

**TABLE 3:** Perceptions of livestock officials on gid and its importance in the livelihood of yak herders

Survey question	Respondents %		
1. How satisfied are you with the National Gid Disease Prevention and Control Program?	Not satisfied	Satisfied	Very satisfied
	39.0 (*n* = 21)	55.0 (*n* = 30)	6.00 (*n* = 3)
2. How serious is the incidence of gid disease in yak rearing areas?	Not serious	Serious	Very serious
	39.0 (*n* = 21)	50.0 (*n* = 27)	11.0 (*n* = 6)
3. Did you know that in absence of veterinarians, yak herders resort to traditionally treating gid?	Yes	No	Don't know
	39.0 (*n* = 21)	28.0 (*n* = 18)	33.0 (*n* = 15)
4. Do you think the herdsmen's practices to control gid are scientifically sound?	Yes	No	Don't know
	0.00 (*n* = 0)	67.0 (*n* = 36)	33.0 (*n* = 18)
5. What could be the reason for not being able to control gid over the years?	Lack of sufficient veterinarians	Lack of policy support	Difficult access to yak areas
	22.0 (*n* = 12)	11.0 (*n* = 6)	67.0 (*n* = 36)
6. Do you think the gid will be controlled fully in the future?	Yes	No	Don't know
	56.0 (*n* = 30)	6.00 (*n* = 3)	38.0 (*n* = 21)
7. Is gid an economically important disease that affects the livelihood of yak herders?	Yes	No	Don't know
	94.0 (*n* = 51)	6.00 (*n* = 3)	0.00 (*n* = 0)
8. How does gid affect the livelihood of yak herders?	Death of calves	Death of adult bulls	Death of milking yaks
	72.0 (*n* = 39)	0.00 (*n* = 0)	28.0 (*n* = 15)
9. What type of herders are most affected by gid?	Herders with big herd size	herders with medium herd size	Herders with small herd size
	72.0 (*n* = 39)	11.0 (*n* = 6)	7.00 (*n* = 4)

### 3.4 Government interventions and impact of gid on herders’ economy

Table 4 presents the households’ responses on awareness programmes, treatment practices of herders in absence of animal health workers, and the impact of gid on herdsmen's economy. A majority of households had not attended awareness programmes organised by the government. It either reflects less interest and low priority of most herders to acquire more knowledge on gid or the busy herding schedule that restricts herders to attend awareness programmes. This has probably created a knowledge gap among herders in better understanding gid, which explains why some herders are skeptical of the advice of animal health workers that dog faeces contribute to gid occurrence in yaks.

**TABLE 4:** Herdsmen's responses on awareness programs, treatment practices of herders in absence of animal health workers, and the impact of Gid on herdsmen's economy

Survey questions	Responses	Household %
Have you attended awareness programs on gid?	Yes	42.6 (*n* = 23)
	No	57.4 (*n* = 31)
	Don't know	0 (*n* = 0)
What are the types of treatment followed for managing Gid during the absence of Animal Health Officials?	Surgery	86.5 (*n* = 47)
	Other treatments	13.5 (*n* = 7)
What is the frequency of visits by Animal Health Official to yak herds?	Never	18.5 (*n* = 10)
	Once annually	66.7 (*n* = 36)
	Twice annually	13.0 (*n* = 7)
	Thrice and more annually	1.80 (*n* = 1)
What is the impact of gid on the local economy?	Highly negative	70.4 (*n* = 38)
	Moderately negative	29.6 (*n* = 6)
	No impact	0 (*n* = 0)

The livestock health worker visited yak herds once annually. A single annual visit to yak herds appears inadequate, as indicated by the continued spread of gid. This is reflected in herders resorting to traditional treatment methods to control gid, in absence of animal health workers. The most common method followed by a vast majority of herders was the surgical operation of infected yaks. Surgery by herders is not a recommended practice, although surgery of heads and brains with cerebral Coenurosis has been reported to be highly successful and effective up to 90% in sheep (Manunta et al., 2012; Scott, 2012). It is surprising to learn that herders performed surgery that could have proved fatal, as surgery is carried out by the trained professionals. Further, personal hygiene and sanitation are rather poor among herders, which likely facilitated the spread of Gid. Without the supervision of animal health workers, the dosage of the anthelmintic drug used for treating the affected yaks remains highly questionable.

The economy of herders depends largely on yak herding. Yak meat and milk products fetch a premium price, and yaks are used to generate cash income during emergencies. A vast majority of respondents mentioned gid as having a highly negative impact on their economy, which reiterates that gid is a major cause of yak mortality in Laya. If continued to flourish, gid could devastate the livelihoods of herders.

## 4 CONCLUSIONS

Gid is not a new disease in yaks. It prevails even today despite several measures to combat the disease. Yak is the livelihood source for mountain communities in Bhutan and it is about time that drastic measures are taken to bring the disease under effective control. The continued menace of gid, as shown by this study, indicates that the National Gid Disease Prevention and Control Plan is yet to be effective. Should gid be allowed to flourish and remain unchecked, the disease could discourage yak herding and accelerate rural out‐migration. Therefore, based on the results of this study, it is recommended that livestock agencies make a renewed approach to creating more awareness on gid, increase the frequency of animal health workers’ visits to yak herds, and increase the frequency of deworming watchdogs and calves.

## DISCLAIMER

The views and interpretations in this publication are those of the authors. They do not imply the expression of any opinion by ICIMOD concerning the legal status of any country, territory, city or area of its authorities, or concerning the delimitation of its frontiers or boundaries, or the endorsement of any product.

## AUTHOR CONTRIBUTION

**Yeshi Wangdi:** Conceptualization; Investigation; Methodology; Resources.

### PEER REVIEW

The peer review history for this article is available at https://publons.com/publon/10.1002/vms3.466.

## Data Availability

The data that support the findings of this study are available from the corresponding author, upon reasonable request.
